# P-1848. Reduction of microbial burden on environmental floor samples through advanced photohydrolysis technology and its impact on *Candida auris*, fungi, and aerobic bacteria

**DOI:** 10.1093/ofid/ofae631.2009

**Published:** 2025-01-29

**Authors:** Kimberly Trosch, Amy Carenza, Deborah Birx, Kirk Huslage, Uzoamaka D Obiekwe

**Affiliations:** ActivePure Technologies, Dallas, TX, USA, Dallas, Texas; ActivePure Technologies, Dallas, Texas; ActivePure Technologies, Dallas, Texas; LifeBridge Sinai Hospital, Baltimore, Maryland; LifeBridge- Sinai Hospital, Baltimore, Maryland

## Abstract

**Background:**

The continued rise of antimicrobial resistance (AMR) and reduction of pharmacological options has created an urgent need for new treatments or countermeasures to combat the spread of multi-drug resistant organisms (MDROs). Focus on MDROs has become paramount with the rise of *Candida auris* (C. *auris*) infections that are associated with high mortality and AMR. Attention must be given to pathogen reservoirs such as the surfaces of floors, which have an underappreciated potential to transfer pathogens to hands from objects in contact with the floor.

**Figure:**
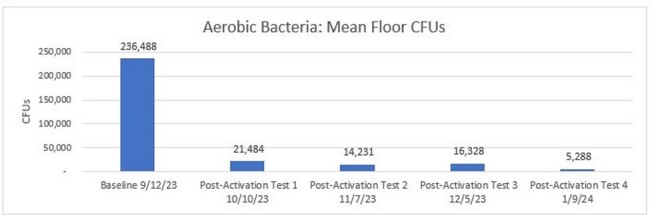

Mean CFUs of Aerobic Bacteria from Baseline to Post-Activation #4.

**Methods:**

An experimental study to explore the impact of advanced photohydrolysis (AP) technology inside a high acuity hospital unit with active C. *auris* infections was performed from September 2023 to January 2024 and investigated the effect of the AP system on floor colony forming units (CFUs) of aerobic bacteria, fungi, and C. *auris*. Baseline pre-activation samples were compared to post-activation samples, which occurred every four weeks on Tuesday mornings prior to shift change and daily environmental services (EVS) cleaning.

**Figure:**
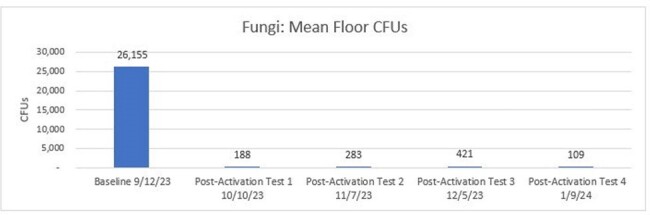

Mean CFUs of Fungi from Baseline to Post-Activation #4.

**Results:**

Pairwise comparisons with a Bonferroni correction found median floor fungal CFUs achieved a statistically significant reduction of 99% (p=.011) from Baseline to Post-Activation #4. Aerobic bacteria decreased 98%; C. *auris* by 66% but did not achieve statistical significance.

The mean CFUs on high-touch surfaces were reduced for aerobic bacteria by 82% and fungi by 99%. C. *auris* was reduced by 100% from Baseline to Post-Activation #3 (41 CFUs to 0 CFUs) but increased in Post-Activation #4 due to two positive sample locations in a patient room with an active C. *auris* infection. Although the locations were positive for C. *auris*, they both tested under 500 CFU/cm^2^, which is associated with a reduced level of infection risk from environmental bioburden.

**Figure:**
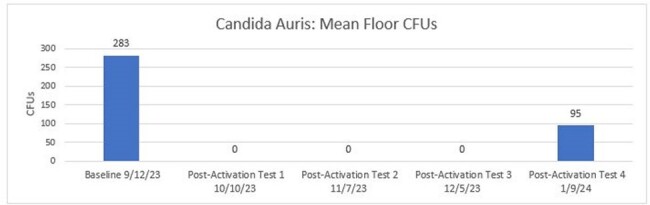

Mean CFUs of Candida Auris from Baseline to Post-Activation #4.

**Conclusion:**

This study clearly demonstrates that despite intensive efforts by EVS, serious microbes remain on floors; however, application of the AP technology provided a continual and persistent decline in floor microbial burden all without the need for additional skilled labor or increases in cleaning and disinfection practices. A forthcoming study will analyze how these demonstrated environmental changes affected patient outcomes.

**Disclosures:**

**Kimberly Trosch, RN, BSN**, ActivePure Technologies: Full time employee **Amy Carenza, BBA**, ActivePure Technologies: Full time employee **Deborah Birx, MD**, ActivePure Technologies: Advisor/Consultant

